# 24-h variations of blood serum metabolites in high yielding dairy cows and calves

**DOI:** 10.1186/s12917-020-02551-9

**Published:** 2020-09-07

**Authors:** Hussein Awad Hussein, Jan-Peter Thurmann, Rudolf Staufenbiel

**Affiliations:** 1 Internal Veterinary Medicine, Department of Animal Medicine, Faculty of Veterinary Medicine, Assiut University, Assiut, 71526 Egypt; 2 Klinik für Klauentiere, Freie Universität Berlin, Königsweg 65, 14263 Berlin, Germany

**Keywords:** Calves, Diurnal, Dairy cows, Metabolic profile, Sampling, Variations

## Abstract

**Background:**

Blood profile testing is commonly used to monitor herd health status, diagnose disorders, and predict the risk of diseases in cows and calves, with subsequent optimization the production of dairy herds. By understanding the physiological ranges of serum metabolites relative to age, lactation stage, and the sampling time in healthy cows and calves, the dairy practitioners can accurately diagnose abnormalities with a blood test. The effect of sampling time on the variation of serum metabolites within 24 h were evaluated in 83 cattle. All animals were originated from a dairy herd, where the animals, based on their ages and lactation stages, were classified into eight groups. The blood samples were collected from each animal every 4 h within a day.

**Results:**

The time of sampling within the day showed significant influences on the serum concentrations of glucose, β-hydroxybutyric acid (BHBA) and urea. BHBA was the most metabolite that showed day variation among cows’ groups. Furthermore, the concentrations of total cholesterol were the most stable metabolite in all groups. The mean values of albumin, total proteins, glucose, non-esterified fatty acids (NEFA), BHBA, total cholesterol, total bilirubin, urea, and creatinine revealed significant variations among the different studied groups.

**Conclusions:**

A certain suitable time of blood sample collection cannot be recommended. However, care shall be taken for the time of sampling for measurements of glucose, NEFA, BHBA and urea, otherwise the comparative values of these metabolites at different sampling time points may differ significantly from each other’s, without a disease cause. It may be recommended, for metabolic assessment of dairy herds, classification the subjects into different groups based on lactation stages and ages of animals.

## Background

Future health and performance management systems should focus on early identification and subsequent prevention of physiological imbalances in dairy herds. Consequently, there is a need for metabolic indicators that reflecting the health status of cows and calves. Measurement of specific serum metabolites allows the evaluation of adequacy of the main metabolic pathways associated with energy, protein, and minerals and then provides useful information related to nutrition and animal health to optimize the productive and reproductive potential of dairy herds [1]. Recently, using of blood metabolic profiling has acquired relevance for the study and diagnosis of various metabolic and reproductive disorders [2]. Blood parameters including glucose, non-esterified fatty acids (NEFA), *β*-hydroxybutyric acid (BHBA), cholesterol, and enzymes and proteins had been reviewed recently to be of great interest for metabolic profiling of dairy herds [3].

Serum metabolites reflecting protein, fat, and carbohydrate metabolism may show a day fluctuation with subsequent difficult interpretations of individual values. Diurnal changes of various blood metabolites have been reported for dairy cows. However, these studies were carried out on few numbers of adult cows [4] or conducted to estimate the effect of feed triglycerides and free fatty acids [5] or administration of monensin [6] on the day variations of blood metabolites and hormones.

Healthy calves form the basis of any successful dairy production system, from both an economic and an animal welfare point of view. However, more research is needed before we can rely exclusively on monitoring data for detection of disease in dairy calves; consequently, routine observation of calves to evaluate metabolic health status remains essential [7]. In addition, characterizing serum metabolites in dairy calves could provide further insight into daily metabolic changes and the mechanisms that lead to metabolic diseases [8]. To the best of authors’ knowledge, the metabolic assessment and the 24-h variations of serum metabolites in newborn and suckling calves, were not yet been investigated.

From the clinical point of view, the strong factors including the age and lactation stage within-day variations of serum metabolites may be a source of misdiagnosis or improper interpretation of the obtained data, therefore, the objectives of this study were as follows: (1) evaluate whether the diagnostic significance of blood metabolites in high yielding cows and calves depends on the time of sampling within 24 h; (2) assess the group variations of different serum metabolites.

## Results

**Fig. 1:**
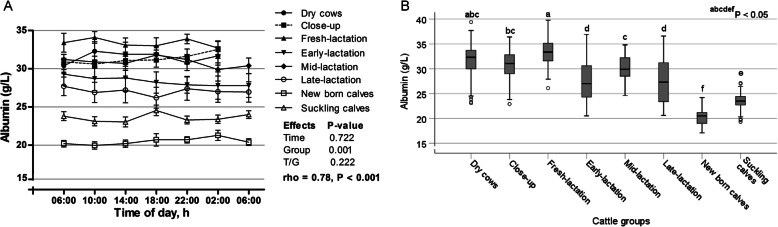
**a** Diurnal variations of serum albumin in dairy cows and calves within 24 h (means±SE). **b** Box plots show the comparison of serum albumin among the different cattle groups. The plots show median (line within box), 25th and 75th percentiles (box), and minimum and maximum values (whiskers). Plots with different letters differ significantly (*P* < 0.05)

**Fig. 2:**
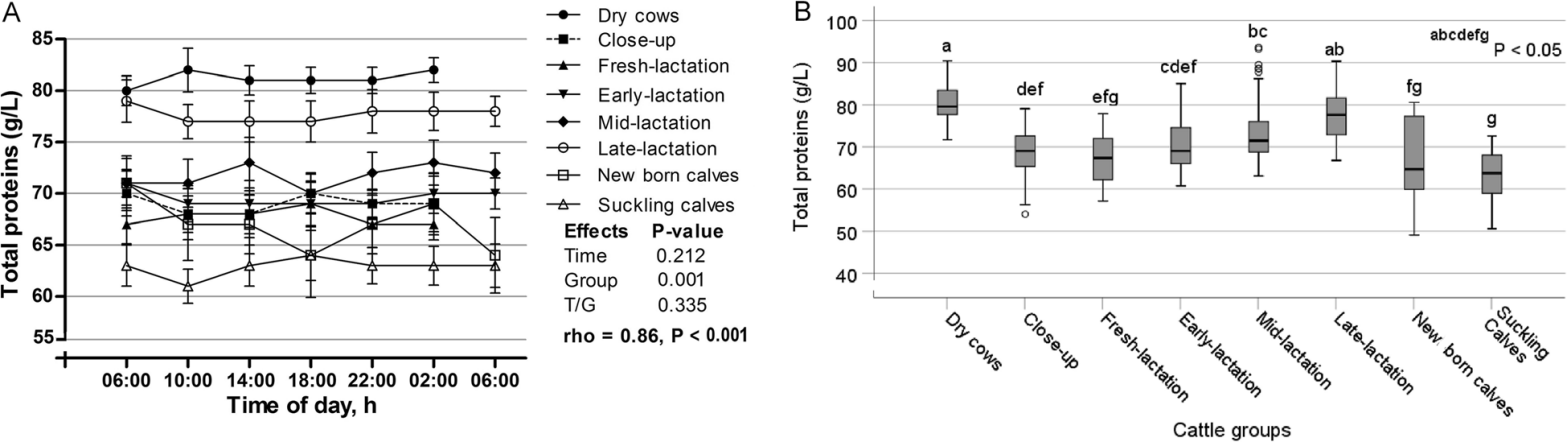
**a** Diurnal variations of serum total proteins in dairy cows and calves within 24 h (means±SE). **b** Box plots show the comparison of serum total proteins among the different cattle groups

The day variations of serum albumin are shown in Fig. 1a. The sampling time has no significant effect on the concentrations of albumin (*p* > 0.05), as well as positive and significant AR1 rho was observed (0.78; *p* < 0.001), suggesting strong relationship among the measurements taken further apart in time. In contrast, the serum levels of albumin were significantly different among the groups (*p* < 0.001); as the highest values were noticed in fresh-lactation cows and the lowest values were observed in newborn calves (Fig. 1b). The day fluctuations of serum total proteins are illustrated in Fig. 2a. The time points showed no significant effect on the values of total proteins (*p* > 0.05), while its level showed significant variations among the different groups (*p* < 0.001), where the group of dry cows exhibited the highest value and suckling calves showed the lowest one (Fig. 2b).

**Fig. 3:**
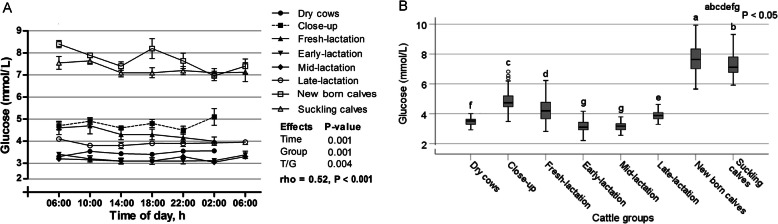
**a** Diurnal variations of serum glucose in dairy cows and calves within 24 h (means±SE). **b** Box plots show the comparison of serum glucose among the different cattle groups

The time relative to sampling has a significant effect on mean glucose levels (*p* < 0.001), as the values varied within the day (Fig. 3a) and regardless the animal groups, the lowest overall mean of glucose was noticed at 02:00 o’clock. There were significant differences in glucose concentrations among groups (*p* < 0.001). However, presence of time/group interaction, indicating that glucose behaved differently with the preceding of sampling time among the groups. The group variations of serum glucose are illustrated in Fig. 3b. The lowest value of glucose was observed in early-lactation cows, while the highest level was noticed in newborn calves (*p* < 0.05).

**Fig. 4:**
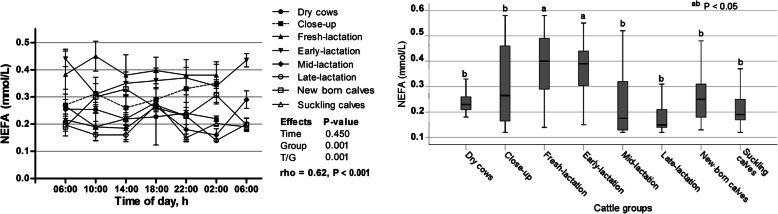
**a** Diurnal variations of serum NEFA in dairy cows and calves within 24 h (means±SE). **b** Box plots show the comparison of serum NEFA among the different cattle groups

Figure 4a illustrates the effect of sampling time during the day, no significant influence on the serum concentrations of NEFA was determined, however, a significant time/group interaction was observed, indicating that NEFA behaved differently within the day among groups. The mean values of NEFA showed significant variations among the different groups (*p* < 0.001), as the highest values were measured in fresh- and early-lactation cows and the lowest were determined in late-lactation ones (Fig. 4b).

**Fig. 5:**
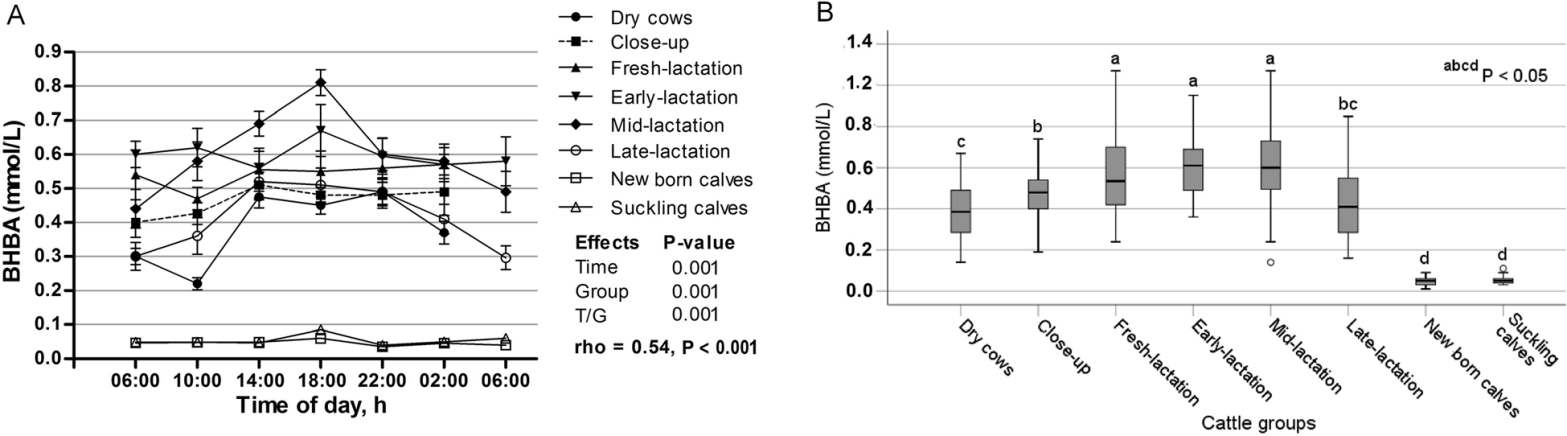
**a** Diurnal variations of serum BHBA in dairy cows and calves within 24 h (means±SE). **b** Box plots show the comparison of serum BHBA among the different cattle groups

The sampling time of the respective day had a significant effect on the concentrations of BHBA (*p* < 0.001; Fig. 5a), as the highest overall mean was observed at 18:00 o’clock. The relationship among measurements taken further apart in time was 0.54 (*p* < 0.001). However, there were significant changes in BHBA levels among different groups (*p* < 0.001). Furthermore, a significant time/group interaction was noticed (*p* < 0.001). Cows of early- and mid-lactation groups showed the highest value of BHBA, while both calves’ groups exhibited the lowest concentrations (*p* < 0.05; Fig. 5b).

**Fig. 6:**
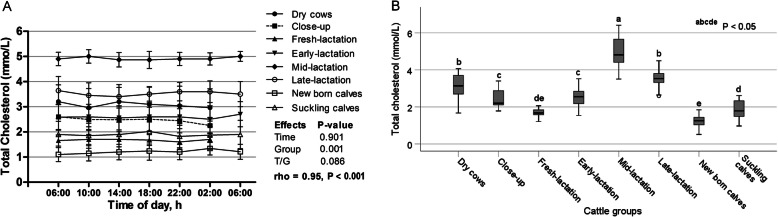
**a** Diurnal variations of serum total cholesterol in dairy cows and calves within 24 h (means±SE). **b** Box plots show the comparison of serum total cholesterol among the different cattle groups

The diurnal fluctuations of serum total cholesterol are shown in Fig. 6a, where no significant effect for the sampling time was noticed (*p* > 0.05). The serum level of cholesterol was highest in mid-lactation cows and lowest in newborn calves (*p* < 0.05; Fig. 6b), with no significant time/group interaction. Furthermore, a positive and strong correlation coefficient was detected (rho = 0.95; *p* < 0.001).

**Fig. 7:**
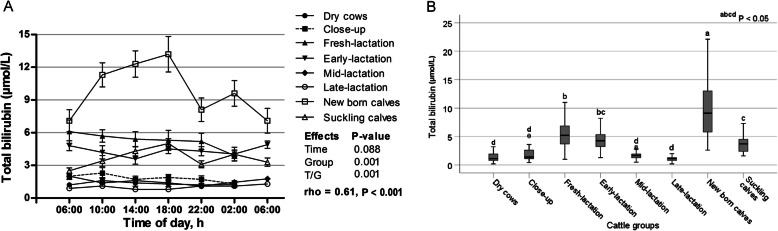
**a** Diurnal variations of serum total bilirubin in dairy cows and calves within 24 h (means±SE). **b** Box plots show the comparison of serum total bilirubin among the different cattle groups

Although the sampling time during the day had no significant influence on the concentrations of total bilirubin (*p* = 0.088), there were significant effects for group and time/group interactions (*p* < 0.001; Fig. 7a). The highest and lowest values for total bilirubin were seen in newborn calves and dry cows, respectively (*p* < 0.05; Fig. 7b). However, because there was a significant time/group interaction for total bilirubin, this metabolite behaved differently among groups within the day.

**Fig. 8:**
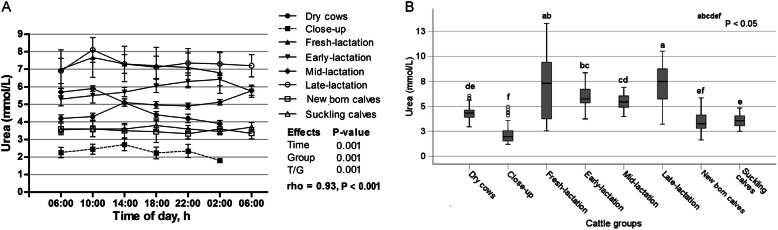
**a** Diurnal variations of serum urea in dairy cows and calves within 24 h (means±SE). **b** Box plots show the comparison of serum urea among the different cattle groups

The time relative to sampling had a significant effect on the mean values of urea (*p* = 0.000), as the values varied within the day (Fig. 8a) and the highest urea concentration was noticed at 14:00 o’clock. There were significant differences in urea concentrations among groups (*p* = 0.000). However, presence of time/group interaction, indicating that urea behaved differently with the preceding of sampling time among the groups. The group changes of serum urea are shown in Fig. 8b. The highest concentration of urea was observed in late-lactation cows, while the lowest value was measured in close-up cows (*p* < 0.05).

**Fig. 9:**
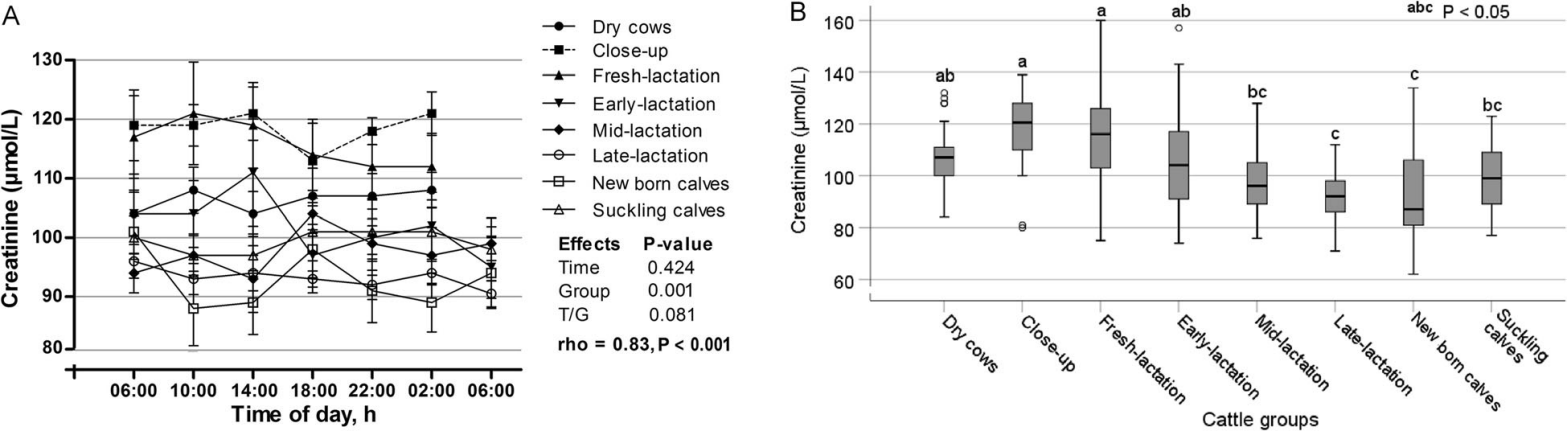
**a** Diurnal variations of serum creatinine in dairy cows and calves within 24 h (means±SE). **b** Box plots show the comparison of serum creatinine among the different cattle groups

The time of sampling during the day had no significant changes on the serum concentrations of creatinine (*p* = 0.424; Fig. 9a). In contrast, the group showed significant effect on the creatinine level (*p* = 0.000) with positive AR1 rho value (0.83; *p* = 000). Cows of close-up phase revealed the highest value, while newborn calves showed the lowest one (*p* < 0.05; Fig. 9b).

## Discussion

In herd management, assessment of the metabolic status have frequently been used to evaluate the nutritional status of dairy cows during the transition period, as well as to monitor the herd health, find subclinical diseases and investigate herd problems with metabolic disorders [9, 10]. In the present study, the influence of strong factors including age (calves, cows), lactation stage (dry period, close-up, fresh lactation, early lactation, mid-lactation, and late lactation) and the time of day (24-h profile) on blood metabolites were investigated.

The sampling time within the day has no significant effect on the serum albumin concentrations, indicating no day dynamic for serum albumin. The albumin concentrations, in the present study, were within the physiological limits and ranged from 30 to 40 g/L [11], with a significant influence for the animal groups on this parameter. Such decrease of albumin concentration in early- and late lactation cows could be explained by the variations of lactation stages. In a previous study [12]; the author attributed the drop of serum albumin after calving to decreased synthesis by the liver and loss from, or dilution in, the blood. The drop of albumin levels in newborn and suckling calves may due to the age factor, as reported elsewhere [13]; calves initially showed decreased albumin concentration 2 days after birth then increased thereafter. Thomas [14] attributed that to increased colostrum uptake with subsequent increased serum globulins. Furthermore, the medium half-life of albumin in ruminant ranges from 14 to 16 days, after that the liver is responsible for albumin synthesis [15].

Within 24 h, the serum concentrations of total proteins were relatively stable without great changes over the time. Supporting this view is that serum proteins may remain reasonably constant over several weeks in an individual, with a coefficient of variation ranged from 3 to 4% [16]. The concentrations of total proteins, in this research, were within the reference values (60–80 g/L) [12]. Conversely, the group showed significant effect on the serum values of total protein, indicating individual variations among the different groups, as it was higher in dry- and late lactation cows, and lower in newborn and suckling calves. The increased serum proteins in dry cows may be explained by the change of metabolic situation after lactation and/or no protein uptake by the mammary gland. It was mentioned before [17], the variation of total protein concentrations during lactation follows a trend comparable with that reported for the variation of milk yield. In addition, Piccione et al. [18] reported that stage of gestation and lactation affected serum total protein concentrations in Holstein-Friesian cows, particularly during the transition from late gestation to early lactation, when cows undergo a pronounced metabolic stress. In contrast, Cozzi et al. [19] did not find any effect of lactation stage on serum total proteins. The lower serum protein concentrations in newborn and suckling calves, in this study, may be explained by low protein contents of drinking milk and/or increased anabolism and consumption of proteins for building up of body musculature. After parturition, the newborn and young calves go through a period of rapid growth, development and major physiological adaptations [20].

The sampling time had a significant effect on the glucose concentration, indicating presence of a day dynamic for this parameter. The drop of serum glucose overnight compared with during the day may be attributed to the utilization and consumption of this metabolite by the different body tissues at that time when the intensity of feed intake is low. This presumptive view was supported elsewhere [6, 21], the authors reported that the feeding activity, the extent of rumen fermentation with high rumen volatile fatty acids, and the availability of substrates for gluconeogenesis were higher during the day than at night. Herbein et al. [22] found reduced night glucose concentrations in dairy cows, whereas another study revealed no diurnal variations for glucose [23]. In contrast, Blum et al. [5] reported increased glucose concentrations during the night. The variations between the different studies are likely to be explained by differences in energy intakes, diet compositions, and time of feedings. In a previous study [24], the authors concluded that the feeding time and feed contents affected the concentration of blood glucose in dairy cows. In newborn and suckling calves, the serum glucose concentrations were higher at the first 06.00 h measurement than at 14.00 and 02:00 h measurements, indicating the diurnal variation of this metabolite, as well as this may be due to the postprandial effect. As the forestomachs of calves, in this study, are not yet developed, therefore the pattern of glucose may seem like that in monogasrtric animals. In monogastrics, it has been reported that minor delays in gastric emptying rate have a notable effect on the glycemic response after a meal [21]. In adult cattle of the current study, only slight glucose fluctuations were found, and the values ​​fluctuated by 3–4 mmol/L with a standard deviation of 0.23–1.1 mmol/L, which may be attributed to the role of insulin in glucose metabolism [25]. It had reported that insulin play an important role in carbohydrate and fat metabolisms leading to lowering of the blood glucose level [12]. In the current study, the serum concentration of glucose showed significant variation among the different groups, as it was higher in newborn and suckling calves, and lower in early- and mid-lactation cows. Such variation may be explained by the change of the metabolic status of each group. Increased glucose level in young calves attributed elsewhere to the composition of milk replacers [26], whereas decreased glucose concentrations in early lactating cows may be as a result of high milk production with subsequent negative energy balance [24].

In the present research, the serum values of NEFA were lower than the critical threshold of 0.57 mmol/L [27]. In addition, the concentrations of NEFA showed insignificant changes during the 24 h measurements, indicating stability of this metabolite with the time of day. However, a significant time/group interaction was noticed, indicating that NEFA might show a day variation in some groups. Blum et al. [5] observed a night rise of NEFA concentration in dairy cows. This difference may be due to the variation of diet contents and time of feed delivery [23]. As reported in a previous study [28], infrequent feed delivery will result in a large variation of feeding behavior throughout the day, resulting in a great change in insulin [29] and, subsequently, fat mobilization with resultant increase of serum NEFA [5]. It was observed a significant effect of groups, in the current work, on the serum concentration of NEFA, as it was higher in fresh- and early lactation cows and lower in late lactation cows. Such significant difference could be explained by the negative energy balance after calving. In early lactating cows, NEFA concentration reflects the fat mobilization for compensation the imbalance between nutrients consumed by the cow and nutrients secreted in milk [30].

The serum concentrations of BHBA showed significant change within the day, indicating variability of this metabolite with the time. Variation within 24 h of serum BHBA was also studied in dairy cows by Blum et al. [5]. In previous studies [23, 29], it was reported that increased serum BHBA after meals was explained by increased conversion of butyrate into BHBA in the ruminal epithelium. All dairy cows, in the current research, were fed either one or two times, but both times were within the day as recommended elsewhere [31], which explains increased BHBA during the day after meals in comparison with the night. The highest BHBA concentrations, in this work, could therefore be observed three to ten hours after feeding. It had been reported increased BHBA concentrations after feedings [6, 23]. The animal groups showed a significant influence on the concentration of BHBA, as it was highest in fresh-, early- and mid-lactation cows. Such increases in these groups could be attributed to the negative energy balance. It is well known that dairy cows at early lactations experience negative energy balance [32]. The lowest values of BHBA were observed among the calves’ groups, which might be explained to the underdeveloped rumen physiology, as well as they were fed energy-containing whole milk drinkers. In dairy calves, the rumen starts in development after 4 weeks of age [33].

In the present study, the cholesterol values showed the most stable course of the day among all examined laboratory parameters. This result was coincided with the observations by Wiedemann et al. [4], who failed to demonstrate 24-h variations for serum cholesterol in bovine animals. In contrast, in a previous study in human [34], the author determined a circadian rhythm with an amplitude of 2.5% in cholesterol daily mean. The cholesterol concentrations showed significant variations among the different groups, as the higher value was noticed in mid-lactation cows. All cows of this group were at the peak of milk production (> 28 kg/day), which might lead to excessive mobilization of lipid to the liver, as sequel of negative energy balance, with subsequent liver sequestration of cholesterol to the blood stream [35]. The lower mean value of cholesterol, in this research, was measured in newborn calves, which may be due to these calves are only drinking whole milk.

In this work, the mean value of total bilirubin in newborn calves was 9.6 μmol/L, above the former mentioned reference value [12]. However, it was stated that serum bilirubin in newborn calves was as high as 9.58 μmol/L [36]. Furthermore, the influence of the time of day could not be statistically verified in the present investigation. However, a significant time/group interaction was detected, indicating that total bilirubin might show a day variation in some groups. Furthermore, the concentrations of total bilirubin in newborn and suckling calves showed the day dynamics. The higher value of this metabolite in the newborn calves may be due to destruction of foetal haemoglobin and slower excretion of bilirubin because of lower concentration of transport protein ligandin, which responsible for passing of indirect bilirubin into hepatocytes where it is transformed to direct bilirubin that can be excreted [12]. In a previous research in calves [37], the authors reported decreased bilirubin concentrations from 1st to the 14th day of age and later it remained stable.

Serum concentrations of urea showed significant variation within 24 h, indicating the day dynamic of this variable, where the highest value was observed at 14:00 o’clock. As all cow groups were supplied with TMR before 14:00 o’clock, which explain the role of feeding behavior on such increase of serum urea. However, calves’ groups showed no day variation of serum urea. In a previous research work [38], the authors contributed the role of meal for the day fluctuation of serum urea concentrations. In contrast to the present work, urea concentrations did not depend on sampling time of the day in dairy cows between week 2 antepartum and week 12 postpartum [4]. Such difference may be attributed to the few number of cows at narrow scale of lactation stages in that study. It was observed a significant effect of groups, in this study, on the serum levels of urea, as the mean value was higher in late lactation cows and lower at close-up ones. Such difference may be explained by the variation of TMR. The reason for the increased urea values in late-lactation cows could be due to the higher protein content of the TMR with a low milk yield (< 28 kg/day). The changes in urea values are affected by protein metabolism and on the other hand by renal excretion of this metabolite [39].

In the present study, serum concentrations of creatinine in all examined groups lie within the specified reference ranges 88–177 μmol/L [11]. No influence for the time of day, in the current investigation, can be proven on serum creatinine concentrations. In contrast, a significant effect for sampling time within the day was observed in sheep [40]. In this study, the serum creatinine concentrations were significantly affected by the animal groups. The higher values were observed in close-up and fresh-lactation cows, indicating the role of calving and its associated physiological adaptation in such increase. The lower values were measured in newborn calves. The reason may be explained by the lower muscle mass than adult cattle. As a product of muscle metabolism, a recent study reported a direct effect for the muscle mass on the urinary excretion of creatinine [41].

## Conclusions

The present study throws a light on the day variations of blood metabolites, which may be helpful for evaluation the health status in high yielding herds. Based on the results, a certain time of blood sample collection cannot be recommended. Therefore, the time of the sample collection can be adjusted according to the operational processes. However, care shall be taken for the time of sampling of glucose, NEFA, BHBA and urea, otherwise the comparative values of the sampling time points may differ significantly from each other, without a disease cause. It may be advisable, for metabolic profiling of high yielding dairy herds, classification of animals into different groups. This classification should be based on the age (young - adult), and lactation stage, to avoid the variation among subjects.

## Methods

The blood sampling procedures reported herein were conducted according to Directive 2010/63/EU, and the regulations of the Institutional Animal Care and Use Committee, Free University of Berlin. All animals were housed, handled and cared according to the rules of German Animal Welfare act (S. 1206, 13,137,833–3/18.05.2006) [42].

### Animals and management

The study was performed on a commercial Holstein-Friesian dairy herd in the city of Leipzig, Germany. During the study period, the herd included 1700 dairy cows. The cows had a 305-d milk yield of 11,500 kg on average (Additional file 1); average milk protein concentration was 3.33% and milk fat was 3.83%. The lactating cows were milked three times per day with 8 h apart. Dairy cows were kept in a free-stall barn equipped with individual electronic feeding systems (Landtechnik Weihenstephan, Germany). Water was available ad libitum from a nose-press water bowl. The dairy calves were raised in individual boxes (igloos) only for about 2 (from 1 to 3) weeks with straw or wood shavings as beddings; they were then housed in-group boxes until the end of the drinking period. A total number of 83 animals were included in the current study. According to the lactation phases and age of animals, they were classified into eight groups as dry cows (*n* = 10), close-up (n = 10), fresh-lactation (*n* = 11), early-lactation (n = 10), mid-lactation (n = 11), late-lactation (n = 11), newborn calves (n = 10) and suckling calves (n = 10). Table 1 summarizes the parity, body condition scores (BCS), investigation stages, and feeding time of studied groups.

**Table 1:** Parity, body condition score (BCS), investigation stages and time of feedings of the study groups

Groups	Parity Mean ± SE (minimum-maximum)	BCS Mean ± SE (minimum-maximum)	Investigation stages	Time of feedings
Dry cows	4.2 ± 0.7 (1–6)	3.1 ± 0.5 (2.5–3.5)	From 2 to 8 weeks’ AP	At 09:30
Close-up	3.1 ± 0.8 (0–4)	3.7 ± 0.3 (3–4)	From 14 to 0 days’ AP	At 10:00
Fresh-lactation	3.8 ± 0.7 (1–6)	3.4 ± 0.6 (3–3.75)	From 1 to 3 days’ PP	At 09:00
Early-lactation	4.1 ± 0.4 (1–6)	3.1 ± 0.4 (2.5–3.5)	From 7 to 30 days’ PP	At 09:00
Mid-lactation	3.7 ± 0.3 (2–5)	2.8 ± 0.3 (2.25–3.25)	From 31 to 150 days’ PP with milk production of ≥28 Kg/day	At 08:00 and 13:00
Late-lactation	4.8 ± 0.6 (2–8)	2.6 ± 0.5 (2–3)	From 174 to 375 days’ PP with milk production of < 28 kg/day	At 07:00
Newborn calves	NA	NA	From 1 to 3 days’ PN	At 03:00 and 15:00
Suckling calves	NA	NA	From 9 to 12 days’ PN	At 03:00 and 15:00

*NA* Not applicable, *AP* Antepartum, *PP* Postpartum, *PN* Postnatal

All animals were clinically examined before and during the study conduction, and all physical parameters including respiratory and heart rates, and rectal temperatures were within the reference ranges. Furthermore, no animals administered any medicaments, just before or during the study conduction, which may interfere with studied metabolites. Cows of all groups were fed diets of grass and maize silage and concentrate as totally mixed rations (TMR) one time per day after the morning milking. However, cows of mid-lactation group received a second feeding after the second milking. The calves were fed whole milk two times per day at 03:00 and 15:00 o’clock; in addition, the newborn calves were given high-quality colostrum containing 50 g immunoglobulins per litre. The stable temperature and air humidity followed the meteorological changes due to the conditions in the stable structure. However, the animals were housed in the stable all over the year with adequate aeration.

### Blood sampling and study design

**Fig. 10:**
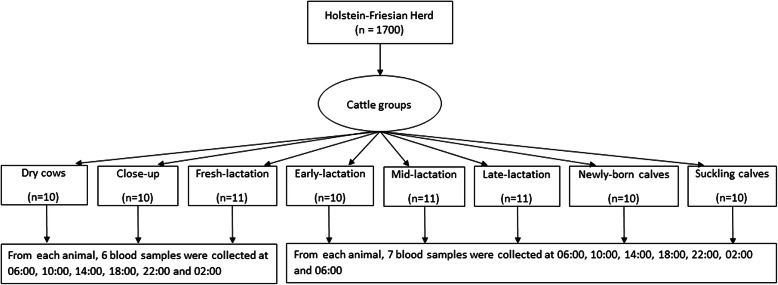
Schematic diagram representing study design and sampling times

In adult dairy cows, blood samples were collected from the tail veins after fixation of cows using 18-gauge cannulas (Sterican, 1,20 × 40 mm, B. Braun Melsungen AG, Melsungen, Germany). In calves, the blood samples were obtained by puncture of jugular vein using catheters (14 G, 2.1 × 50 mm, Braun, Germany), which were fixed in the skin after shaving and disinfection. All blood samples were collected in 9 ml vacutainer tubes without anticoagulant (Monovet® 9 ml Z, SARSTEDT AG & Co., Nümbrecht, Germany). Blood samples were taken at 4 h interval over 24 h period, starting at 06:00 o’clock, and ending at 06:00 o’clock of the following day, indicating 7 blood samples were collected from each animal. Except in dry, close-up and fresh-lactation cows, blood collection started at 06:00 o’clock and ended at 02:00 o’clock of the following day, indicating 6 blood samples were obtained only from each cow (Fig. 10).

Blood samples were kept at room temperature for about 30 min to clot. Subsequently, samples were centrifuged at 2000×*g* for 20 min to harvest serum samples (Heraeus Sepatech Labofuge 200, Heraeus Holding GmbH, Hanau, Germany). Serum samples were harvested into 5 ml tubes (Röhrchen, SARSTEDT AG & Co., Nümbrecht, Germany), using an automatic pipette (Transferpipette 3,5 ml, SARSTEDT AG & Co., Nümbrecht, Germany), and frozen at − 18 °C until biochemical assays of albumin, total proteins, glucose, NEFA, BHBA, total bilirubin, cholesterol, urea and creatinine.

### Laboratory assays

The biochemical analyses were carried out in the laboratory of Ruminant Clinic, Free University of Berlin. The serum concentrations of albumin and total proteins were determined using Biuret and Bromocresol green methods, respectively. The measurement of glucose was done using the hexokinase method. A commercial analysis colorimetric method (Randox Laboratories Limited) was used for the quantitative serum determination of NEFA. A quantitative kinetic enzymatic method (Ranbut RB 1007; Randox Laboratories Limited) was used for measurement of BHBA. The determination of total bilirubin was done using a quantitative method (Jendrassik-Gróf). The analysis of cholesterol was performed using a quantitative method (CHOD-PAP). The concentrations of urea and creatinine were measured using enzymatic and kinetic assays, respectively. The sensitivity and coefficient of variation for measurements of 9 studied blood metabolites were provided (Additional file 2). All laboratory assays were carried out automatically using the Roche Cobas Mira Plus CC Chemical Analyzer (Roche Diagnostics, Bern, Switzerland).

### Statistical analysis

Statistical analyses were performed with IBM SPSS Statistics (Version 25, Munich, Germany). The first step is the detection of outlier data for each blood metabolite and values more than 3 standard deviations away from the mean were discarded. No more than 3% of data were removed from any single measurement. For statistical evaluation, a repeated measures analysis of variance was calculated using the linear mixed procedure, where the animal numbers were the subject, time relative to sampling was set as a repeated variable, and autoregressive [AR(1)] was used as a covariance structure. The model included terms for time relative to sampling, animal groups, and time x group interaction. The following linear mixed model was used for analysing the effects of sampling time, animal groups and their interactions: *M*_*jk*_ *= μ + T*_*j*_ *+ G*_*k*_ *+ TG*_*jk*_ *+ ε*_*jk*_*.*

Where *M* = the observed level of serum metabolites, *μ* = the overall mean, *Tj* = the fixed effect of sampling time (*j* = 06:00, 10.00, 14:00, 18:00, 22:00, 02:00, 06:00), *G*_*k*_ = fixed effect of the cattle groups (*k* = 1, 2, 3, 4, 5, 6, 7, 8), TG_jk_ = Timej and Groupk interaction, ε_jk_ = residual error. The main effects were tested using least square differences (LSD). Effects were considered significant at *p* < 0.05. In each animal group, the day variations for each metabolite are illustrated as line diagrams (means ±SE). For comparison among the different animal groups, the mean value of each metabolite was used, and the data are shown as boxplot illustrations.

## Supplementary information

**Additional file 1.** The amount of milk in kg. (average = 11,500 kg/305 d, *n* = 1700 cows).

**Additional file 2.** Sensitivity and coefficient of variations for assays of the 9 blood metabolites (clinic laboratory).

## Data Availability

The datasets generated and/or analyzed during the current study are available from the corresponding author on reasonable request.

## Abbreviations

BHBA: *β*-hydroxybutyric acid
NEFA: Non-esterified fatty acids
