# Supplementary material for: 24-h variations of blood serum metabolites in high yielding dairy cows and calves
Source: BMC Vet Res. 2020 Sep 7;16:327. doi: 10.1186/s12917-020-02551-9 (PMC7487511; doi:10.1186/s12917-020-02551-9)
Supplement: Supplementary file 1 — Additional file 1. The amount of milk in kg. (average = 11,500 kg/305 d, n = 1700 cows). [file 12917_2020_2551_MOESM1_ESM.docx]

Supplementary file 1

The amount of milk in kg. (average = 11500 kg/305 d, n = 1700 cows)

|  | n | Mean | SD | Min | Max | x_25_ | x_50_ | x_75_ |
| --- | --- | --- | --- | --- | --- | --- | --- | --- |
|  |  |  |  |  |  |  |  |  |
| Colostrum^1^ | 1549 | 5,4 | 3,43 | 0 | 23,5 | 3 | 4,8 | 7 |
| MD 2^2^ | 1525 | 26,4 | 8,78 | 3 | 56 | 20 | 27 | 33 |
| MD 3^2^ | 1525 | 28,8 | 9,29 | 3 | 56 | 22 | 30 | 36 |
| MD 4^2^ | 1523 | 31,0 | 9,71 | 3 | 60 | 24 | 32 | 38 |
| MD 5^2^ | 1524 | 32,9 | 10,27 | 3 | 62 | 25 | 34 | 41 |
| MD 6^2^ | 1522 | 34,3 | 10,53 | 3 | 65 | 26 | 35 | 42 |
| MD 7^2^ | 1517 | 35,7 | 10,65 | 3 | 64 | 28 | 37 | 44 |
|  |  |  |  |  |  |  |  |  |
| TM 1^3^ | 1507 | 189,3 | 55,9 | 24 | 333 | 145 | 198 | 232 |
|  |  |  |  |  |  |  |  |  |
| MW 1^4^ | 1507 | 31,6 | 9,32 | 4 | 55,5 | 24,2 | 33 | 38,7 |
| MW 3^4^ | 1357 | 39,1 | 10,26 | 6 | 63 | 31 | 41 | 47 |
| MW 4^4^ | 1349 | 40,9 | 10,52 | 3 | 67 | 32 | 43 | 49 |
| MW 5^4^ | 1353 | 41,9 | 10,69 | 5 | 67 | 33 | 44 | 50 |
| MW 6^4^ | 1337 | 42,5 | 10,79 | 7 | 76 | 33 | 44 | 50 |
| MW 7^4^ | 1326 | 43,0 | 10,50 | 7 | 73 | 34 | 45 | 51 |
|  |  |  |  |  |  |  |  |  |
| MP 100^5^ | 1260 | 4066 | 924,4 | 1640 | 6310 | 3273 | 4190 | 4798 |
|  |  |  |  |  |  |  |  |  |
|  |  |  |  |  |  |  |  |  |

MD, Milking day; TM, Total milk; MW, Milking week; MP, Milk production

^1^ First day colostrum

^2^ Amount of milk on milking days from 2 to 7

^3^ Total amount of milk in the first week of lactation

^4^ Daily milk amount in lactation weeks from 2 to 7

^5^ 100-day-Milk production
