# Supplementary material for: 24-h variations of blood serum metabolites in high yielding dairy cows and calves
Source: BMC Vet Res. 2020 Sep 7;16:327. doi: 10.1186/s12917-020-02551-9 (PMC7487511; doi:10.1186/s12917-020-02551-9)
Supplement: Supplementary file 2 — Additional file 2. Sensitivity and coefficient of variations for assays of the 9 blood metabolites (clinic laboratory). [file 12917_2020_2551_MOESM2_ESM.docx]

Supplementary file 2

Sensitivity and coefficient of variations for assays of the 9 blood metabolites (clinic laboratory)

| Parameters | Sensitivity | Intra-assay CV^a^  in % (n) | Inter-assay CV^1^  in % (n) | Inter-assay CV^2^  in % (n) |
| --- | --- | --- | --- | --- |
| Total proteins | 2,0 g/l | 1,8 (10) | 2,7 (21) | 2,8 (21) |
| Albumin | 2,87 g/l | 0,5 (10) | 2,6 (20) | 3,0 (20) |
| Urea | 0,33 mmol/l | 3,2 (10) | 4,6 (16) | 3,0 (16) |
| Cholesterol | 0,08 mmol/l | 1,1 (10) | 2,7 (16) | 2,8 (16) |
| Bilirubin | 1,71 µmol/l | 2,6 (10) | 2,1 (21) | 3,9 (21) |
| Glucose | 0,1 mmol/l | 1,4 (10) | 3,9 (13) | 5,7 (14) |
| NEFA | 72 µmol/l | 3,9 (10) | 3,4 (34) | 3,9 (35) |
| BHBA | 0,1 mmol/l | 3,2 (10) | 11,6 (31) | 7,2 (30) |
| Creatinine | 2,65 µmol/l | 3,9 (10) | 2,8 (10) | 2,0 (10) |

^a^ coefficient of variation

^1^ physiological range, n single repeat measurements over a period of 4 months

^2^ pathological range, n single repeat measurements over a period of 4 months

Sensitivity and coefficient of variations for assays of the 9 blood metabolites; information were obtained from the testkit manufacturers (Randox), two or three series (n = 20)

| Parameters | Sensitivity | Intra-assay CV  in % | Inter-assay CV  in % |
| --- | --- | --- | --- |
| Total proteins | 2,0 g/l | 0,65; 0,73; 0,75 | 1,36; 1,43; 1,57 |
| Albumin | 2,87 g/l | 3,99; 4,52 | 0,71; 1,28 |
| Urea | 0,33 mmol/l | 1,50; 2,37; 4,52 | 1,88; 3,08; 6,08 |
| Cholesterol | 0,08 mmol/l | 0,55; 0,62; 1,32 | 1,06; 1,38; 1,93 |
| Bilirubin | 1,71 µmol/l | 1,34; 2,41; 2,74 | 1,91; 2,85; 4,12 |
| Glucose | 0,1 mmol/l | 2,11; 2,11; 2,21 | 0,79; 0,91; 0,94 |
| NEFA | 72 µmol/l | 4,74; 4,81 | 4,32; 4,51 |
| BHBA | 0,1 mmol/l | 3,76; 3,78 | 5,06; 5,25 |
| Creatinine | 2,65 µmol/l | 0,69; 1,30; 4,05 | 1,27; 2,37; 5,21 |
